# Perioperative NSAIDs and Long-Term Outcomes After cancer Surgery: a Systematic Review and Meta-analysis

**DOI:** 10.1007/s11912-021-01133-8

**Published:** 2021-11-08

**Authors:** Shebin Shaji, Charlotte Smith, Patrice Forget

**Affiliations:** 1 School of Medicine, Medical Sciences and Nutrition, University of Aberdeen, Foresterhill Health Campus, Aberdeen, AB25 2ZD UK; 2 Department of Anaesthesia, NHS Grampian, Aberdeen, UK

**Keywords:** NSAIDs, Perioperative, Cancer, Disease-free survival, Long-term outcomes, Surgery

## Abstract

**Purpose of Review:**

This review investigated the use of perioperative non-steroidal anti-inflammatory drugs (NSAIDs) and long-term outcomes in cancer surgery patients, and whether this is dependent on cancer type, type of NSAID and timing of administration.

**Findings:**

Perioperative NSAID use was found to be associated with longer disease-free survival (hazard ration, *HR* = 0.84 (95% CI, 0.73–0.97)) and overall survival (*HR* = 0.78 (95% CI, 0.64–0.94)). No difference was found between different types of NSAID for disease-free survival, although in overall survival ketorolac use was significant (*HR* = 0.63 (95% CI, 0.42–0.95)). Analysis on the timing of NSAID administration found no subgroup to be associated with cancer outcomes. The cancer-type analysis found an association with outcomes in breast and ovarian cancers. However, the level of certainty remains very low, mostly due to the heterogeneity and the retrospective nature of most studies.

**Summary:**

Perioperative NSAID use may be associated with increased disease-free and overall survival after cancer surgery. This may be dependent on the type of cancer and type of NSAID, and further research is needed to support this. These data may inform future prospective trials, which are needed to determine the clinical impact, as well as optimal NSAID regimen.

## Introduction

Although surgical resection is a mainstay of curative cancer treatment, surgery has been identified as a high-risk time for cancer progression [1]. During surgery, an increased number of circulating tumour cells (CTCs) has the potential to travel to distant sites and form “micrometastases” [2]. This may be aided by increased vascularity, due to an increase in angiogenic factors [3, 4], and immune suppression, mediated by pain and neural activation [5, 6]. Surgical stress also leads to local and systemic inflammation [1, 7]. This heightens the risk of recurrence, as environments rich in inflammatory molecules are more susceptible to colonisation by CTCs [8].

Preclinical and clinical studies have suggested that interventions given during the perioperative period greatly influence cancer recurrence and survival [9, 10]. This fragile period provides an opportunity to tip the balance between pro- and anti-metastatic signals and potentially determine whether cancer progresses or regresses [11, 12]. Non-steroidal anti-inflammatory drugs (NSAIDs) are one method which could be utilised in the anaesthetic regimen to interrupt the surgical stress response. NSAIDs are known to inhibit the cyclooxygenase (COX) isoenzymes, which convert arachidonic acid to prostaglandins [13, 14]. Excess production of prostaglandin has been shown to be key in various oncological events [15].

Recent literature has evidenced that NSAIDs are beneficial in cancer treatment and prevention [16–18]. Further studies have also shown that NSAIDs can reduce the production of angiogenic factors [19–21]. However, the long-term use may negatively impact on protective COX mechanisms [22] so a short course of NSAIDs around the time of surgery may maximise benefit whilst limiting harm [1]. Importantly, animal studies have shown continuous and perioperative use of NSAIDs to reduce metastases by the same degree [23].

A human study found that patients receiving a postoperative NSAID plus fentanyl had decreased serum concentrations of the inflammatory markers VEGF, TNF-α and IL-1β when compared to fentanyl alone [24]. However, these studies did not investigate survival outcomes, leaving the clinical benefit unclear. Even a small benefit could be significant due to the relatively safe nature and cost-effectiveness of the NSAID drug class [25].

This review and meta-analysis investigated the use of perioperative NSAIDs on cancer patients that underwent surgical intervention with intention to cure. It examined any association with disease-free survival (DFS) and overall survival (OS). A meta-analysis was conducted to further investigate whether the type of cancer, type of NSAID and timing of administration influenced these associations. These important variables were investigated with the aim of identifying the right NSAID, the right patients and the right timing for optimum effect.

## Methods

A systematic review was conducted in line with the protocols established in the Preferred Reporting Items for Systematic Reviews and Meta-Analyses (PRISMA) [26] and the Cochrane Handbook for Systematic Reviews of Interventions version 6.1 [27]. Protocols were registered on the Prospero website on 05/02/21 detailing early search strategy and eligibility criteria. Database searches were carried out on Ovid Medline (13/01/2021), Cochrane database (14/01/2021) and www.clinicaltrials.gov (14/01/2021) with an example search strategy available in Appendix 1.

### Study Screening

Inclusion and exclusion criteria for the review were established according to the PICOS method (Population, Intervention, Comparison, Outcomes and Study design) suggested by the PRISMA guidelines: Population (P): cancer patients undergoing surgery with curative intent; Intervention (I): perioperative NSAIDs; Comparison (C): control patients that received no NSAIDS; Outcomes (O): long-term survival, cancer recurrence, overall survival; Study Design (S): human subjects, English language. Exclusion criteria included reviews, case reports, letters, ongoing trials, trials with no results, single-arm studies with no comparative group and short-term outcome measurement.

Rayyan QCRI software was used to export search results [28]. Two reviewers, independently screened titles and abstracts against eligibility criteria. Any conflicts during the process were discussed and resolved by a third party where agreement could not be reached.

### Data Synthesis

Hazard ratios with 95% confidence intervals were extracted for DFS and OS outcomes and data were aggregated using the Cochrane RevMan5 software [29]. Subgroup analyses were also performed according to the predefined research questions. Sufficient data were available to compare types of NSAIDs used in the studies, cancer type and timing of administration. A narrative synthesis was performed for dose and duration.

### Risk of Bias Analysis

Scottish Intercollegiate Guidelines Network (SIGN) checklists for cohort and randomised control trials [30] were used to assess the risk of bias within the included studies. Randomised control trials were further assessed using the Cochrane Risk of Bias tool. These were used to determine the potential for selection bias, detection bias and others. Finally, the GRADE classification system [27] was used to determine the likelihood that the interventions used were causally linked with the outcomes of focus.

## Results

### Search Results and Study Selection

**Fig. 1:**
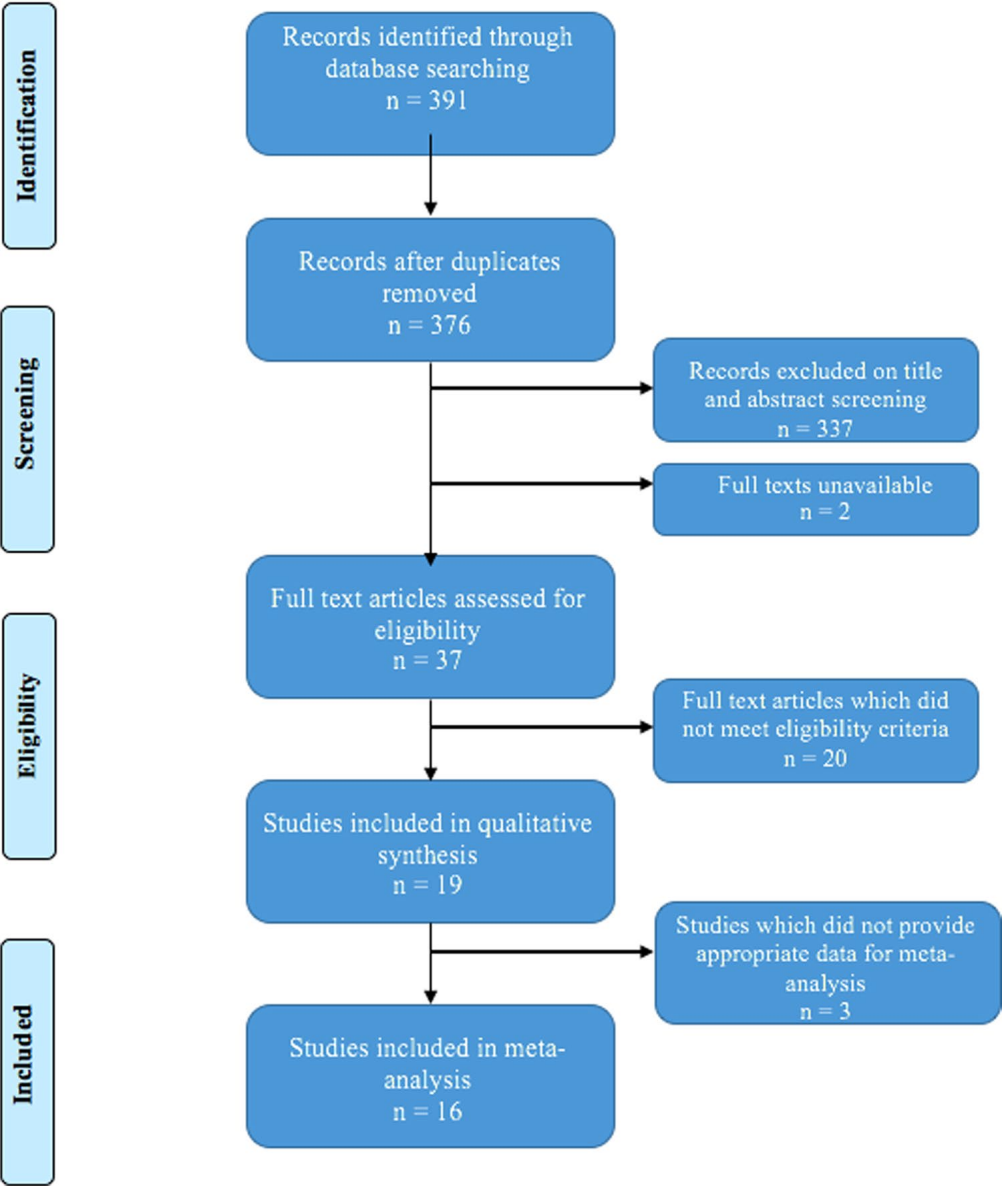
Flow diagram illustrating the number of studies included in each stage of the literature search. Adapted from PRISMA guidelines [26]

The results of the search strategy are summarised in the PRISMA flow diagram in Figure 1. Ovid Medline search produced 255 studies; Cochrane database produced 52; and www.clinicaltrials.gov produced 84. Twenty studies were found to fit the eligibility criteria; however, no contact was received from authors of Lee et al. [31] regarding data needed, so the remaining 19 were included. Of these, 16 provided sufficient data to be included in the meta-analysis.

**Fig. 2:**
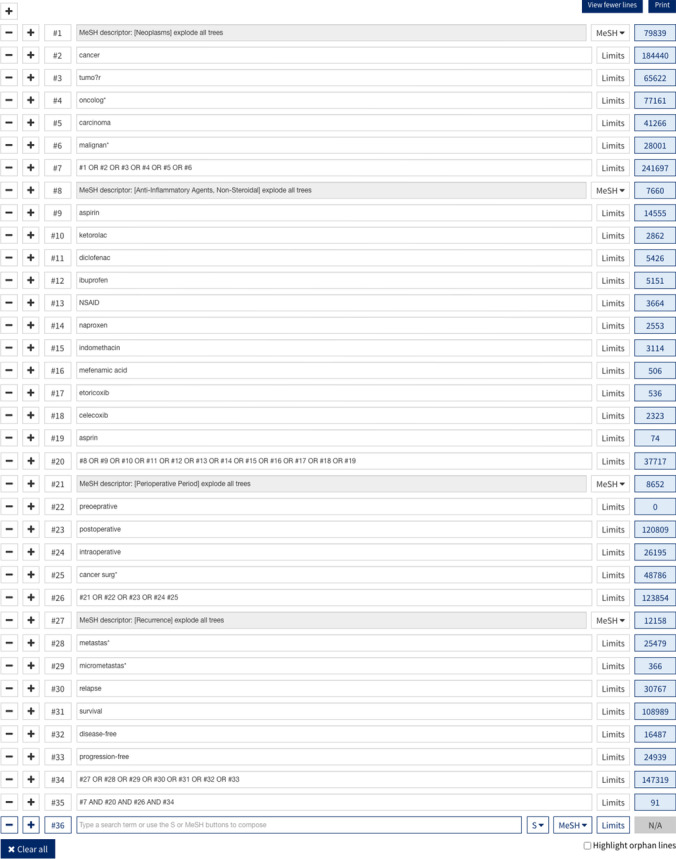
Search Strategy Example - Cochrane Database, searched 14/01/2021

During the screening process, there were thirteen conflicts between the two reviewers. These were resolved through discussion, and seven conflicts which could not be resolved were assessed by a third reviewer who made the final decision. The inter-rater variability test was performed and showed 96.5% concurrence between the two reviewers Fig. 2.

### Study Characteristics

Data were extracted from 19 studies with a total of 12994 participants. Study design and characteristics are summarised in Table 1.

**Table 1:** Characteristics of the included studies

Study	Study design	Number of participants	Cancer type	NSAID(s) used	Dosage	Timing of administration	Comparator	Outcomes measured
Cho [49]	Prospective randomised control trial	50	Breast cancer	Ketorolac	Ketorolac: 60 mg	Intraoperative (end of surgery)	Propofol-ketorolac vs. sevo-fentanyl	Immune and inflammatory response (NK cells, IL-2, neutrophils, lymphocytes, WCC), cancer recurrence or metastasis
Choi [38]	Retrospective cohort study	1139	Non-small cell lung cancer	Ketorolac, ibuprofen, celecoxib	Multiple dosages given	Postoperative	No NSAIDS	Recurrence-free survival; overall survival
de Castro Araujo [32]	Retrospective cohort study.	516	Melanoma	Tenoxicam, ketorolac, parecoxib	Single dose, not specified	Intraoperative	No NSAIDS	Time to treatment failure, recurrence-free interval; melanoma-specific survival; disease-free survival; overall survival
Desmedt [50]	Retrospective cohort study	1834	Breast cancer	Ketorolac, diclofenac	Ketorolac: single dose of 20 or 30 mg depending on weight, diclofenac: 75 mg	Intraoperative	No NSAIDS	Distant recurrence
Forget [39]	Retrospective cohort study	327	Breast cancer	Ketorolac, diclofenac	Ketorolac: single dose of 20 or 30mg depending on weight. Diclofenac: 50mg 3 times daily for three days	Intraoperative ketorolac, postoperative diclofenac	No NSAIDS	Recurrence-free survival
Forget [44]	Retrospective cohort study	1111	Prostate cancer	Ketorolac	24 mg (± 7)	Intraoperative	No NSAIDs	Biochemical recurrence-free survival
Forget [40]	Retrospective cohort study—4 centres	Breast—357 Lung—255 Kidney—227	Breast cancer, lung cancer, kidney cancer	Ketorolac, diclofenac	Ketorolac: single dose of 20 or 30 mg depending on weight, diclofenac: 75 mg	Intraoperative (some beginning, some end of surgery)	No NSAIDs	Prognostic significance of NLR value, recurrence-free survival, overall survival
Forget [33]	Retrospective cohort study	720	Breast cancer	Ketorolac, diclofenac	Ketorolac: single dose of 20 or 30 mg depending on weight, diclofenac: 75 mg	Intraoperative (preincisional)	No NSAIDs	Prognostic significance of NLR value, disease-free survival, overall survival
Forget [34]	Prospective, double-blind, randomised control trial	203	Breast cancer	Ketorolac	Ketorolac: single dose of 30 mg	Intraoperative (preincisional)	No NSAIDs	Disease-free survival, secondary malignancy, overall survival, distant met-free survival
Goh [41]	Retrospective cohort study	726	Colorectal cancer	Aspirin	Not specified	Multiple time points	No NSAIDs	Disease-specific survival, recurrence-free survival, overall survival
Guo [47]	Retrospective cohort study	123	Ovarian cancer	Ketorolac	Ketorolac 15 or 30 mg depending on creatinine clearance	Multiple time points	No NSAIDs	Disease-specific survival
Huang [35]	Retrospective cohort study	515	Rectal cancer	Not specified	Not specified	Multiple time points	No NSAIDs	Prognostic significance of PLR, recurrence-free survival, disease-free survival, overall survival, anastomotic complications
Jiang [37]	Retrospective cohort study	347	Non-small cell lung cancer	Indomethacin, ibuprofen	Indomethacin: 75 mg 4× daily or 25 mg 3× daily, ibuprofen: 200 mg 3× daily	Postoperative (within 48 h of surgery)	No NSAIDs	Progression-free survival, overall survival
Lee [42]	Retrospective cohort study	1637	Non-small cell lung cancer	Ketorolac alone or in combination with ibuprofen, rofecoxib or celecoxib	Ketorolac: 30–60 mg/day Ibuprofen: 200–800 mg/day Rofecoxib: 50 mg/day Celecoxib: 200–300 mg	Postoperative (within 72 h of surgery)	No NSAIDs	Recurrence-free survival, overall survival, PLR
Lönnroth [48]	Prospective randomised control trial	28	Colorectal cancer	Indomethacin, celecoxib	Indomethacin: 50 mg 2× daily, celecoxib: 40 mg 2× daily + esomeprazole prophylaxis	Preoperative (72 h prior to surgery)	Sham control—esomeprazole prophylaxis	Expression of prominin 1, disease-specific survival
Mao [43]	Retrospective cohort study	185	Bladder cancer	Parecoxib	Parecoxib: 40-mg single dose	Intraoperative	No NSAIDs	Recurrence-free survival, overall survival
Schack [36]	Cohort study based on prospective data	2308	Colorectal cancer	Ibuprofen, diclofenac, others	Ibuprofen: at least 800 mg/day, diclofenac: at least 50 mg/day	Postoperative (minimum of 2-day treatment within 7 days of surgery)	No NSAIDs	Recurrence, disease-free survival, 5-year mortality
Wuethrich [45]	Retrospective cohort study	261	Prostate cancer	Ketorolac	Ketorolac: 30 mg	Postoperative (every 8 h for 48 h from surgical closure)	Thoracic epidural anaesthesia without NSAIDs	Biochemical recurrence-free survival, clinical progression-free survival, cancer-specific survival, overall survival
Wuethrich [46]	Retrospective cohort study	148	Prostate cancer	Ketorolac	Ketorolac: 30 mg	Postoperative (every 8 h for 48 h from surgical closure)	Thoracic epidural anaesthesia without NSAIDs	Biochemical recurrence-free survival, clinical progression-free survival, cancer-specific survival, overall survival

### Risk of Bias

The risk of bias in each study is summarised in Appendix 2, with a later discussion of the risk of bias in each outcome.

### Study Outcomes

#### DFS

All nineteen studies looked at long-term oncological outcomes, as per the PICO criteria. Five studies stated “disease-free survival” as an outcome [32, 33, 34••, 35•, 36•] whilst a further one stated “progression-free survival” [37] and six “recurrence-free survival” [38–43]. Despite differing terms, these outcomes were sufficiently similar when defined in their respective studies to be considered in the overall DFS analysis. This included the three prostate cancer studies [44–46] for which biochemical recurrence-free survival was included in the DFS analysis, as PSA above 0.2 ng/ml was the apparent measure for recurrence. Two studies which looked at disease-specific survival [47, 48] and two which looked at distant recurrence were also included [49, 50•]. Regarding Forget et al. [40], data for RFS were extracted, except for the lung cancer centre where distant-metastasis-free survival was used.

Hazard ratios for DFS were extracted from twenty cohorts and aggregated into a forest plot. Where the studies had calculated hazard ratios for subgroups as well as for overall NSAID vs. control comparison, overall use was extracted for this comparison. Some of the studies subcategorised data either by centre [40] or by type of NSAID used [39, 50•], and so these hazard ratios were extracted and included as separate cohorts in the final meta-analysis.

Thirteen cohorts reported a hazard ratio less than 1 which favoured the NSAID group compared to the non-NSAID group on disease-free survival. Seven of these cohorts had a statistically significant *p* value of less than 0.05. Seven cohorts had a hazard ratio of greater than 1 favouring the non-NSAID group; however, none of them was statistically significant.

#### OS

Twelve studies stated “overall survival” as an outcome [32, 33, 34••, 35•, 37, 40–43, 45, 46] with one further study looking at 5-year mortality, defined as death by any cause, which was sufficiently similar [36•]. These were all included in the secondary outcome analysis. Hazard ratios were extracted from thirteen cohorts for OS and aggregated into a forest plot.

Nine cohorts reported a hazard ratio below 1 which favoured the NSAID group over the non-NSAID group in OS; however, only four of these values are statistically significant with a *p* value of less than 0.05. Four cohorts had a hazard ratio of greater than 1 and none of these groups had a statistically significant *p* value.

Meta-analyses showed an association with both DFS and OS outcomes in the groups that received perioperative NSAIDs (Appendix Figures 3 and 4). However, heterogeneity was high, especially in DFS where *I*^2^ = 60%, indicating a significant level of inconsistency between studies. Forest plots can be found in Appendix 3 and results are summarised in Table 2.

Three studies were not included in the meta-analysis, as they did not provide hazard ratios for DFS or OS. Their findings are briefly summarised:

Cho et al. [49] found no association between NSAID use and DFS. Lee et al. [42] also found no statistically significant difference in DFS or OS between the NSAID group and control. Lönnroth et al. [48] found that disease-specific survival was 522 days ± 107 vs 313 ± 106 in the sham control group. No statistical analysis was performed on these numbers.

**Table 2:** Overall effect estimates for each outcome in the comparison between perioperative NSAID use and no perioperative NSAID use (adapted from Cochrane handbook template for “Summary of Findings Table” [27])

Outcome	Effect estimate (HR)	95% confidence intervals	No. of participants (studies)	Certainty of the evidence (GRADE)	Comments
*Disease-free survival*	0.84	0.73–0.97	11075 [16]	**Low ⊕⊕⊝⊝** Due to the risk of bias in studies, high heterogeneity, and imprecise definitions	*Perioperative NSAIDs may be associated with increased disease-free survival.*
*Overall survival*	0.78	0.64–0.94	6954 [11]	**Low ⊕⊕⊝⊝** Due to the risk of bias in studies	*Perioperative NSAIDs may be associated with increased overall survival.*

### Cancer-Type Subgroup

Data were aggregated into forest plots and stratified by cancer type, which can be found in Appendix 4. In DFS, heterogeneity was high in the breast cancer and lung cancer subgroups, with *I*^2^ = 77% and *I*^2^ = 57%, respectively. A statistically significant association with longer DFS was only seen in the breast cancer subgroup and ovarian cancer subgroup, although ovarian cancer had only one study so no aggregation was possible. For lung, colorectal and prostate cancer, aggregation detected no significant association between NSAID use and DFS. Bladder cancer and melanoma also had only one study each which meant that the aggregate was not applicable.

Seven of the cohorts did not report OS hazard ratio. In the OS analysis, NSAID use in none of the cancer types was significantly associated with OS. Heterogeneity was lower with breast cancer *I*^2^ = 41%, lung cancer *I*^2^ = 45%, and prostate cancer *I*^2^ = 40%, although the number of included studies was also lower. Ovarian, tongue and kidney cancer did not report hazard ratios for OS so were excluded from this sub-analysis.

### Type of NSAID Subgroup

Two studies found an association with better cancer outcomes in perioperative ketorolac use, but not in diclofenac use [39, 50•]. Choi et al. [38] also found perioperative ketorolac use to be an independent predictor of OS in early-stage lung cancer, but not ibuprofen or celecoxib. However, a prospective randomised control trial looking at ketorolac use found no statistical difference in DFS or OS when compared to patients who had not received ketorolac [34••]. Meta-analysis was performed to look at the effects of each drug type across studies.

Eleven studies which investigated a single type of NSAID, or disaggregated data for individual types of NSAID, gave hazard ratios for DFS and were included in a forest plot for DFS [36•, 39–41, 43–47, 50•]. Six also calculated hazard ratios for OS for their respective NSAID and were therefore included in secondary outcome analysis [34••, 36•, 40, 43, 45, 46]. Forest plots can be found in Appendix 5.

The remaining studies grouped patients who had received multiple types of NSAIDs and consequently could not be used in this meta-analysis.

For the primary outcome, DFS, all NSAID-type subgroups crossed the line of no effect. This means there was no statistically significant difference between NSAID and control. Heterogeneity remained high in this subgroup analysis, with *I*^2^ = 59% in the ketorolac studies. Overall, the hazard ratios which most favour the NSAID were seen in ketorolac, although the association was not found to be statistically significant.

For the secondary outcome, OS, the only type of NSAID found to have a statistical effect in comparison with control was ketorolac (*HR* = 0.63 (95% CI, 0.42–0.95)). Heterogeneity was lower in the ketorolac studies in the OS analysis, with *I*^2^ = 28%, but the number of studies included was also lower. Heterogeneity remained high in the diclofenac studies with *I*^2^ = 58%.

### Timing

Goh et al. [41] found that preoperative aspirin users had worse RFS, whereas the risk of recurrence and death in postoperative aspirin users was approximately 60% lower compared to patients who had not used postoperative aspirin. Forget et al. [39] found an association between intraoperative NSAID use and improved DFS, but not postoperative, although different drugs were used at each time point.

Meta-analysis was performed to compare time points. Studies were excluded from this subgroup analysis that were not specific about timings or which grouped patients who received NSAIDs at multiple time points.

For DFS, fourteen studies reported hazard ratios [32–34••, 36•, 37–40, 43–46]. Only one study provided sufficient data for inclusion in the preoperative use subgroup [41]. For OS, no studies had calculated hazard ratios for preoperative use, so this subgroup could not be included in the secondary outcome analysis. However, five could be included in the intraoperative subgroup [32–34••, 40, 43] and five in the postoperative subgroup [36•, 37, 38, 45, 46]. Both data sets were pooled in forest plots, which can be found in Appendix 6.

For both DFS and OS, there was no difference detected between intervention and control for either intraoperative or postoperative NSAID subgroups. Preoperative use was associated with a worsened DFS in the single study which was included in this analysis. A larger degree of heterogeneity was seen in the data from intraoperative studies, with *I*^2^ = 53% compared to *I*^2^ = 35% in the DFS analysis.

### Dose and Duration

In studies which used ketorolac, eight included participants who had been given a single dose [33, 34••, 39, 40, 44, 47, 50•]. Only the prospective trials were able to be specific about dose, with Cho et al. [49] giving 60 mg and Forget et al. [34••] giving 30 mg. The rest were retrospective and so included patients whose doses varied depending on patient factors such as weight and creatinine clearance. This meant that a comparison of dosage was not plausible. Three studies looked at multiple doses of ketorolac [42, 45, 46]. None of these found a statistically significant difference between NSAID and control use.

In diclofenac, three studies used a single intraoperative dose of 75 mg [33, 40, 50•]. Desmedt et al. [50•] found no association with DFS and Forget et al. [40] found a positive association with DFS in their lung cancer cohort. The third study did not disaggregate data for diclofenac so conclusions could not be drawn. A further two studies included multiple doses [36•, 39]. Neither study found an association with the cancer outcomes investigated by this review.

The remaining NSAIDs used were not comparable in terms of dose and duration because doses or timings were not specific or only a single study looked at that particular drug.

## Discussion

### Interpretation of Results

#### Overall NSAID Effect

The current review looked at 12,994 participants who were treated perioperatively with NSAIDs. Meta-analysis showed an association between perioperative NSAID use and longer DFS and OS. This suggests that a short course of NSAIDs in the perioperative period may be beneficial for cancer surgery patients. However, there is a considerable amount of heterogeneity between the studies, and most trials were based on retrospective data, indicating that this association could be due to other factors.

#### Cancer-Type Subgroup

The average HR of all breast cancer studies indicated that NSAIDs given to breast cancer patients may reduce the risk of disease recurrence by 42%. However, the heterogeneity was very high. A strength of this subgroup analysis was that this type of cancer had the most studies and data recorded which improved precision. This analysis had a low heterogeneity score which added confidence to the result.

The average HR of DFS for lung cancer is optimistic as this analysis detected a 31% chance of reduction in disease recurrence. However, wide confidence intervals and the important heterogeneity preclude any definitive conclusion. There were only three study data available which was also a limitation.

The average HR for DFS in colorectal cancer indicated that there was no difference between NSAIDs and non-NSAIDs group. Zero percent heterogeneity between the studies added strength to the aggregate value. Three out of the four cohorts were from the same study which possibly reduces the reliability of the result.

There was no effect observed for prostate cancer patients. More studies investigating this effect should be conducted to corroborate this no effect, as COX 2 is overproduced in prostate cancer cells, and studies have shown that COX2 inhibitors can induce apoptosis in prostate cancer cell lines [51–53]. Another weakness in the analysis of the result is that Forget et al. [44] had a relatively short follow-up period with a mean of 38 months. Therefore, these results could be different with a longer follow-up, as prostate cancer patients live for many years after diagnosis which can make interpretation of survival outcomes problematic.

The association between perioperative NSAID use and longer DFS in breast cancer surgery is consistent with many of the breast cancer studies which is reassuring for clinicians and patients. In contrast, other cancer types such as prostate, colorectal, bladder and melanoma did not appear to be affected. This could imply that the effect of NSAIDS is tumour specific, with host immune responses being altered differently by NSAID mechanisms in different cancer types.

Since only one study investigated bladder cancer, ovarian cancer and melanoma, firm conclusions are difficult to draw. However, the results do suggest that there was a benefit in bladder cancer and ovarian cancer. This indicates a demand for more observational studies or prospective studies in these less common cancers.

#### Type of NSAID Subgroup

Meta-analysis indicated that ketorolac may have the greatest effect on oncological outcomes. However, if the association between ketorolac and improved OS was due to the proposed anticancer effects of NSAIDs, then it would be expected that this would also affect DFS, which, in this analysis, was not seen.

On balance, fewer studies reported OS, which could have underpowered the analysis and may therefore account for the different results. This discrepancy could also be explained by the retrospective designs of many of the included studies. Retrospective cohort studies were more likely to have differing participant characteristics between intervention and control groups, so there may have been confounding factors which affected OS but not DFS in some of the studies. For example, several studies noted that younger patients were more likely to receive NSAIDs [32, 33]. This means that a detected OS benefit could be age related rather than intervention related. Additionally, only one study was included for each of parecoxib, aspirin and ibuprofen, affecting certainty regarding the accuracy of these results.

For these reasons, it cannot be definitively concluded that any particular NSAID is superior in effect on oncological outcomes. Nevertheless, the trends identified by this analysis may be useful in the design of future studies. Ketorolac could be the NSAID most likely to give favourable outcomes and would therefore be a good candidate for future prospective trials.

### Timing of NSAID Subgroup

No statistical effect was detected for intraoperative or postoperative NSAID subgroups. Preoperative NSAID use was found to negatively impact on survival outcomes; however, only one study provided hazard ratios for preoperative use, so this result was underpowered.

This subgroup analysis was limited by varying definitions of the terms “intraoperative” and “postoperative”. For intraoperative studies, all were included that stated “intraoperative” as the timing of administration. Yet, some were specific to induction of anaesthesia, some were given pre-incision and others were given prior to surgical closure. These studies were grouped together for the purposes of this meta-analysis as per the protocol. In the “postoperative” subgroup, some studies gave a single postoperative dose, others multiple doses within 72 h and others included patients who received NSAIDs for longer. For example, Schack et al. [36•] included NSAID use of at least 2 days within the first 7 days after surgery. As NSAIDs are thought to inhibit surgery-induced inflammation, some of these NSAIDs may have been administered too late to have any impact on outcomes. Consequently, some of these specific time points may be superior to others, but this analysis would have been unable to detect this.

### Dose and Duration

Due to inconsistency between studies, it was difficult for this review to draw conclusions on optimal dose or duration. In ketorolac use, more favourable outcomes were seen in studies which looked at a single dose rather than multiple doses, where no effect was seen. However, the timings of these doses could be responsible for this trend, rather than dosage, as single doses were all intraoperative and multiple doses all postoperative. Dosage also appeared to be dependent on patient factors such as weight and renal function. Further research will be required to identify any dose-dependent difference in outcomes.

## Review Strengths and Limitations

This is the most updated systematic review exploring whether perioperative NSAIDs can improve DFS and OS for cancer patients. This review was strengthened by the protocol which was registered with Prospero prior to formal searching. Searching multiple databases attempted to ensure no relevant studies were missed. Predefined PICO criteria reduced the risk of bias in study selection as did the use of two team members independently screening and appraising the studies. This is an independent systematic review with no conflicts of interest minimising additional bias.

Fifteen studies were retrospective which carry an inherent risk of selection bias due to the absence of randomisation. Furthermore, pooling data from observational retrospective studies put the results at a risk of residual confounding. Some studies did not report certain confounding variables such as BMI status and smoking status. These are crucial factors that could have affected OS and DFS. The high heterogeneity can also create management when interpreting the results. In addition, not all studies reported complete data required for analysis. This could also have introduced a risk of publication bias if the unpublished data were not favourable towards the intervention and consequently not reported. The fact that most studies included multivariate adjustments reduced the effect of residual confounding and adds more confidence in the results.

These studies could also carry a risk of detection bias, as follow-up and outcome measurement cannot be predefined in a retrospective study. However, this was minimised as survival outcomes were largely objective. Many centres also used published methods or validated algorithms for detecting recurrence or distant metastases, strengthening the consistency between studies.

Aggregating data for outcomes which varied in definition was a limitation of this review. DFS takes into account distant metastases, locoregional recurrence and disease-specific survival, whereas some included studies only took one or two of these factors into account in their definitions. Others looked at each of these factors separately. These outcome definitions may have masked true effects across studies; however, definitions were sufficiently similar to identify overall trends which could inform future trials.

## Further Considerations

Some included studies also looked at the use of NSAIDs specifically in patients who had high levels of inflammatory markers. This is rational as these patients are more at risk of dormant tumour cells being activated due to environments rich in inflammatory cells. Huang et al. [35•] did not find a significant association between perioperative NSAID use and DFS overall, but did find a significant association in a subset of patients with high PLR. Two studies also found that high NLR levels were an independent predictor of poor survival [33, 40], highlighting the potential importance of these inflammatory markers. Targeting perioperative NSAID therapy to these particular subgroups may be an important development in cancer surgical outcomes.

## Conclusion

Surgical stress may increase the risk of postoperative cancer recurrence or metastases. Anaesthetic interventions may have a role in preventing this process to improve oncological outcomes. NSAIDs may be an important factor, as this review has found an association between perioperative NSAID use and longer DFS and OS. This review has also given clues as to the right cancer type, the right drug and the right timing for administration in order to maximise this effect. Even a small benefit may be significant due to the safety profile and cost-effectiveness of this class of drugs. However, more prospective trials are required to confirm the best anti-inflammatory regimen.

## Figures and Tables

**Table 3:**
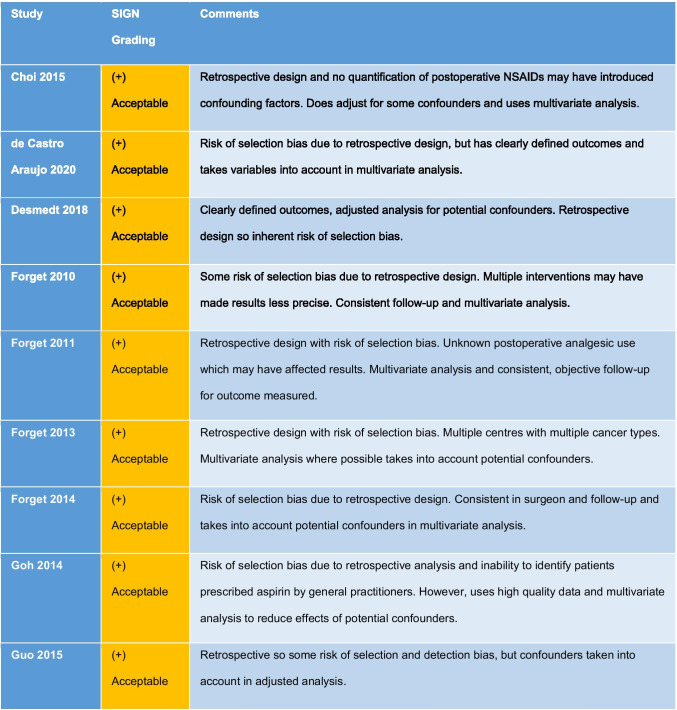
Risk of bias in included cohort studies. Risk according to SIGN guideline methodology assessment for cohort studies, with comments on potential sources of bias within each study. Retrospective studies could not be graded higher than “acceptable” according to SIGN guidelines

**Table 3:**
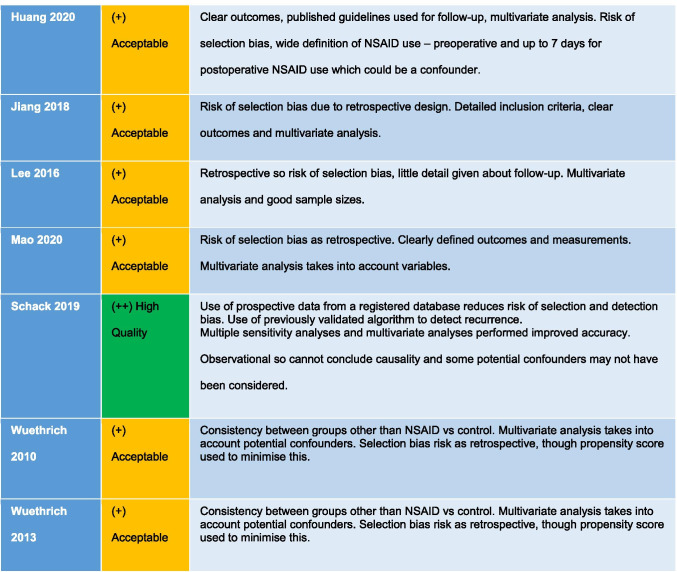
(continued)

**Table 4:**
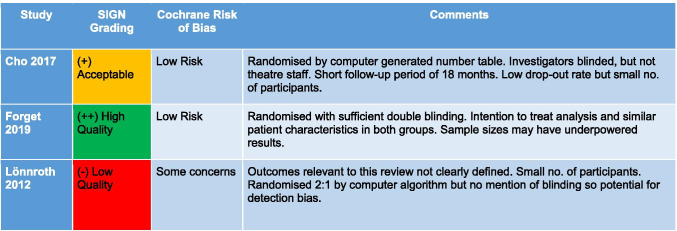
Risk of bias in included randomised control trials. Risk according to SIGN guideline methodology assessment for randomised control trials, and Cochrane risk of bias tool, with comments on potential sources of bias within each study

**Fig. 3:**
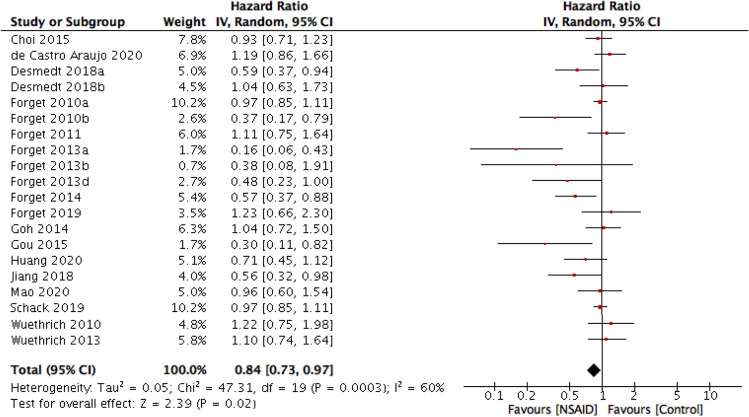
Comparison of disease-free survival in any perioperative NSAID use vs. no perioperative NSAID use. Studies included are those that provided hazard ratios for disease-free survival, with 95% confidence intervals. Desmedt 2018a = ketorolac; Desmedt 2018b = diclofenac; Forget 2010a = diclofenac; Forget 2010b = ketorolac; Forget 2013a = breast centre 1; Forget 2013b = breast centre 2; Forget 2013d =lung centre.

**Fig. 4:**
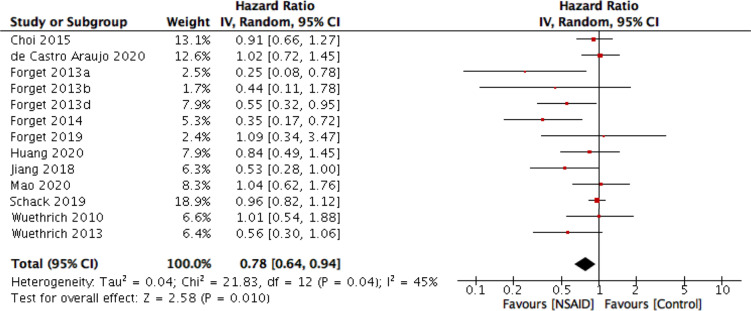
Comparison of overall survival in perioperative NSAID use vs. no perioperative NSAID use. Studies included are those that provided hazard ratios for overall survival, with 95% confidence intervals. Forget 2013a = breast centre 1; Forget 2013b = breast centre 2; Forget 2013d =lung centre

**Fig. 5:**
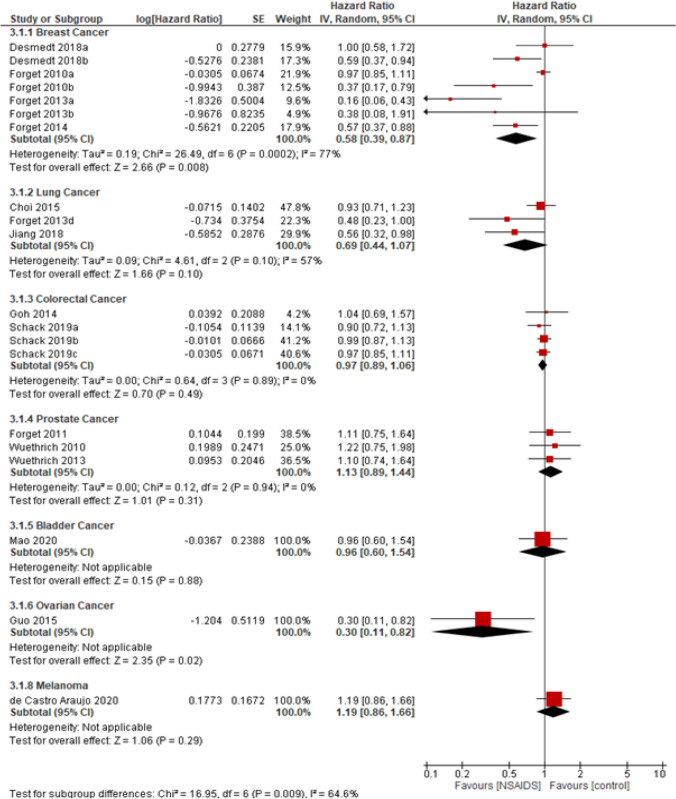
shows DFS stratified by type of cancer. Desmedt 2018a = ketorolac; Desmedt 2018b = diclofenac; Forget 2010a = diclofenac; Forget 2010b = ketorolac; Forget 2013a = breast centre 1; Forget 2013b = breast centre 2; Forget 2013d = lung centre; Schack 2019a = diclofenac; Schack 2019b = ibuprofen; Schack 2019c = overall NSAID group.

**Fig. 6:**
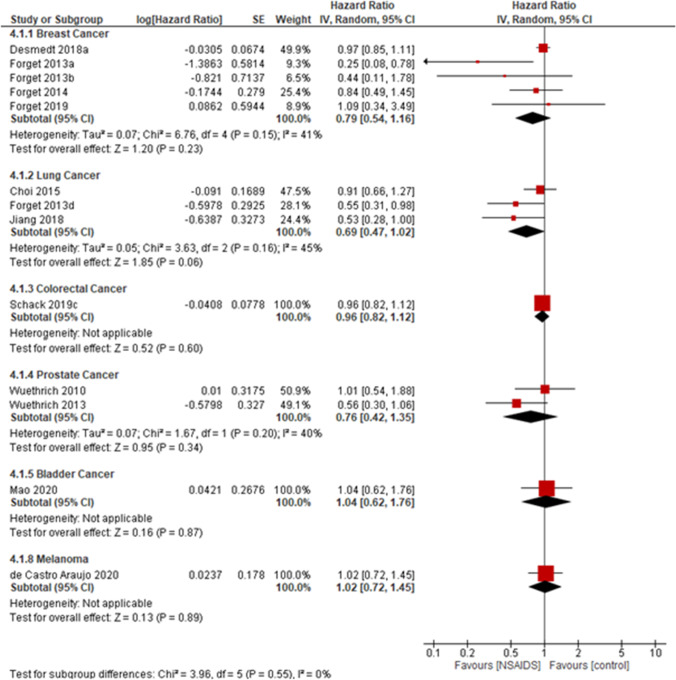
shows OS stratified by type of cancer. Desmedt 2018a = ketorolac; Forget 2013a = breast centre 1; Forget 2013b = breast centre 2; Forget 2013d = lung centre; Schack 2019c = overall NSAID group.

**Fig. 7:**
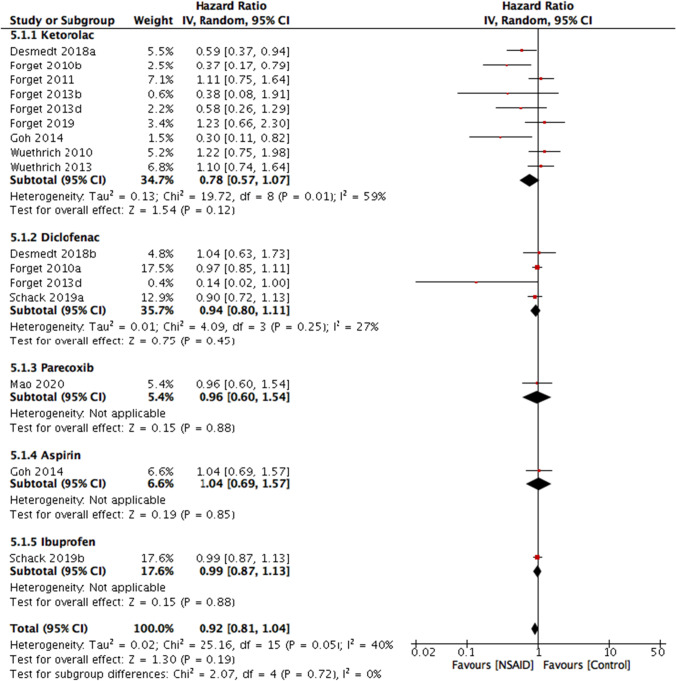
Comparison of disease-free survival in perioperative NSAID use vs. no perioperative NSAID use, grouped by type of NSAID given. Studies included are those that provided hazard ratios for disease-free survival, with 95% confidence intervals, in a specific named NSAID drug. Desmedt 2018a = ketorolac; Desmedt 2018b = diclofenac; Forget 2010a = diclofenac; Forget 2010b = ketorolac; Forget 2013b = breast centre 2; Forget 2013d = lung centre; Schack 2019a = diclofenac; Schack 2019b = ibuprofen

**Fig. 8:**
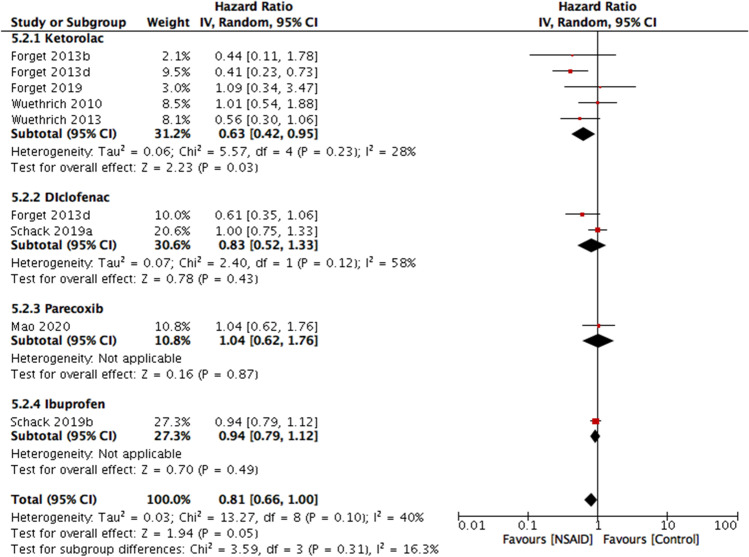
Comparison of overall survival in perioperative NSAID use vs. no perioperative NSAID use, stratified by type of NSAID. Studies included are those that provided hazard ratios for overall survival, with 95% confidence intervals, in a specific named NSAID type. Forget 2013b = breast centre 2; Forget 2013d = lung centre; Schack 2019a = diclofenac; Schack 2019b = ibuprofen

**Fig. 9:**
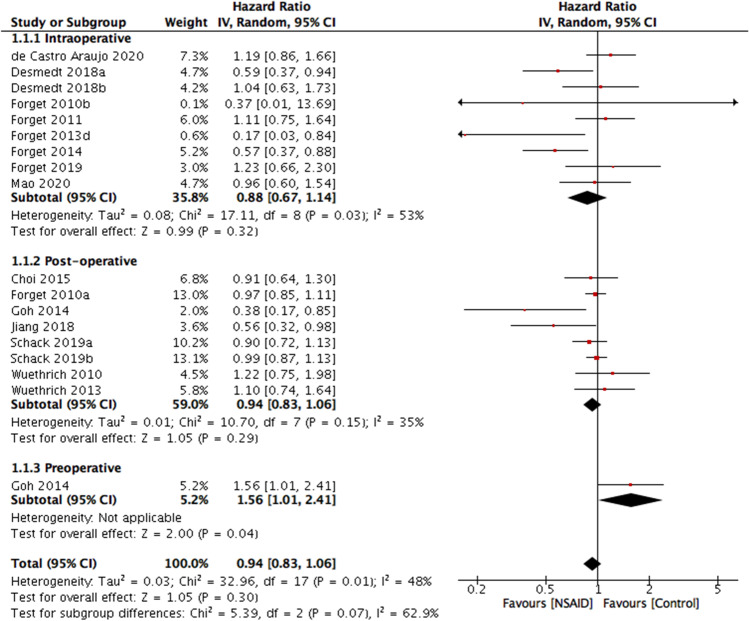
Comparison of disease-free survival in NSAID use vs. no NSAID use, grouped by the timing of NSAID administration. Studies are grouped to compare intraoperative, postoperative, and preoperative use. Studies included are those that provided hazard ratios for disease-free survival, with 95% confidence intervals, and gave NSAIDs at specific time points only, or investigated specific time point subgroups. Desmedt 2018a = ketorolac; Desmedt 2018b = diclofenac; Forget 2010a = diclofenac; Forget 2010b = ketorolac; Forget 2013d = lung centre; Schack 2019a = diclofenac; Schack 2019b = ibuprofen

**Fig. 10:**
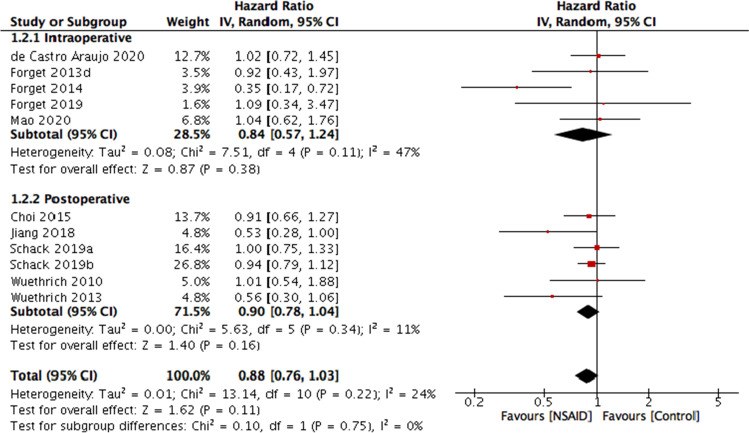
Comparison of overall survival in NSAID use vs. no NSAID use, grouped by the timing of NSAID administration. Studies are grouped to compare intraoperative use with postoperative use. Studies included are those that provided hazard ratios for overall survival, with 95% confidence intervals, and gave NSAIDs at specific time points only, or investigated specific time point subgroups. Forget 2013d = lung centre; Schack 2019a = diclofenac; Schack 2019b = ibuprofen.

## Appendix 2

Tables 3 and 4

## Appendix 3

Meta-analysis of Perioperative NSAIDs

Fig. 3

Fig. 4

## Appendix 4

### Cancer-Type Subgroup Analysis

Fig. 5

Fig. 6

## Appendix 5

### Type of NSAID Subgroup Analysis

Fig. 7

Fig. 8

## Appendix 6

### Timing of NSAID Administration Subgroup Analysis

Fig. 9

Fig. 10
